# A predictive model for early recurrence of colorectal-cancer liver metastases based on clinical parameters

**DOI:** 10.1093/gastro/goaa092

**Published:** 2021-01-26

**Authors:** Siqi Dai, Yao Ye, Xiangxing Kong, Jun Li, Kefeng Ding

**Affiliations:** 1 Department of Colorectal Surgery, The Second Affiliated Hospital, Zhejiang University School of Medicine, Hangzhou, Zhejiang, P. R. China; 2 Cancer Institute (Key Laboratory of Cancer Prevention and Intervention, China National Ministry of Education, Key Laboratory of Molecular Biology in Medical Sciences, Zhejiang Province, China), The Second Affiliated Hospital, Zhejiang University School of Medicine, Hangzhou, Zhejiang, P. R. China

**Keywords:** colorectal cancer, liver metastases, early recurrence, prediction model, post-operative surveillance

## Abstract

**Background:**

The prognosis for patients with colorectal-cancer liver metastases (CRLM) after curative surgery remains poor and shows great heterogeneity. Early recurrence, defined as tumor recurrence within 6* *months of curative surgery, is associated with poor survival, requiring earlier detection and intervention. This study aimed to develop and validate a bedside model based on clinical parameters to predict early recurrence in CRLM patients and provide insight into post-operative surveillance strategies.

**Material and methods:**

A total of 202 consecutive CRLM patients undergoing curative surgeries between 2012 and 2019 were retrospectively enrolled and randomly assigned to the training (*n *=* *150) and validation (*n *=* *52) sets. Baseline information and radiological, pathological, and laboratory findings were extracted from medical records. Predictive factors for early recurrence were identified via a multivariate logistic-regression model to develop a predictive nomogram, which was validated for discrimination, calibration, and clinical application.

**Results:**

Liver-metastases number, lymph-node suspicion, neurovascular invasion, colon/rectum location, albumin and post-operative carcinoembryonic antigen, and carbohydrate antigen 19–9 levels (CA19–9) were independent predictive factors and were used to construct the nomogram for early recurrence after curative surgery. The area under the curve was 0.866 and 0.792 for internal and external validation, respectively. The model significantly outperformed the clinical risk score and Beppu’s model in our data set. In the lift curve, the nomogram boosted the detection rate in post-operative surveillance by two-fold in the top 30% high-risk patients.

**Conclusion:**

Our model for early recurrence in CRLM patients after curative surgeries showed superior performance and could aid in the decision-making for selective follow-up strategies.

## Introduction

Colorectal cancer (CRC) ranks as the world’s third most common cancer and ranks second in cancer-related mortality [1]. In China, CRC is the fifth leading cause of cancer-specific mortality and the third in annual incidence [2]. The incidence of metastatic CRC is fairly high: a quarter present metastasis upon diagnosis, while a half develop metastasis later during the course of the disease [3, 4]. The liver is the most frequent site of metastasis [5]. For resectable CRC liver metastases (CRLM), hepatic excision is considered the most effective method to reach potential cure and long-term survival [6, 7]. Radiofrequency ablation (RFA) therapy, owing to its simple, repeatable, and low-risk nature [4], is popularized in unresectable CRLMs [8–10]. Moreover, multidisciplinary management and the introduction of neoadjuvant treatment are rapidly altering the landscape of CRLM therapy [11, 12]. Unfortunately, although no-evidence-of-disease (NED) could be achieved in a number of patients, there exists great heterogeneity in their prognosis. Some could enjoy quality NED survival, while many develop CRC recurrence early after curative operation [13–15].

Early recurrence (ER), defined as tumor relapse within 6* *months of curative surgery for CRLM [13, 16], is significantly associated with worse gross tumor behavior and poor prognosis [17–19]. Post-operative surveillance should be individualized. For patients who are highly likely to develop ER, more radical surveillance strategies are critical for timely detection and intervention, instead of a routine check-up every 3–6 months after curative surgeries [20, 21]. Studies on liquid biopsy [15, 22, 23] and gene profiling [24] in predicting ER are rather preliminary and financially draining, and are miles away from routine practice. In addition, their clinical accuracy was not adequately presented. On the other hand, the predictive effect of a single clinical [25, 26] or radiological [27] parameter is also limited due to the complexity of CRLM patients’ condition. Medical practitioners now lean more on experience rather than objective predictive tools. Thus, developing a predictive system that provides accuracy, usability, and cost-effectiveness is of critical importance.

Herein, we aimed to develop and validate a comprehensive nomogram utilizing multiple clinical characteristics to predict ER after curative surgeries in CRLM patients and provide insight into post-operative surveillance strategies.

## Patients and methods

### Patients

Between June 2012 and December 2019, consecutive CRLM patients who underwent resection of the primary site and hepatic excision/RFA for liver metastases with curative intention in our institution (the Second Affiliated Hospital of Zhejiang University School of Medicine) were retrospectively enrolled in this study and randomly assigned to the training and validation sets. The inclusion criteria were as follows: signed written consent; CRC patients with synchronous or metachronous liver metastases; patients who underwent single-stage or two-stage surgeries with curative intent; adenocarcinoma as the pathological type; and with or without neoadjuvant chemotherapy. The exclusion criteria were as follows: recurrent CRLM; remnant lesions confirmed by post-operative radiological/ultrasound examination; noncompliance with routine post-operative surveillance; lost to follow-up; or incomplete medical record. The terminal event was ER, defined as a compromise of NED with radiological confirmation due to relapse of CRC (regional or distal) within 6 months after curative surgery where the state of NED was realized [13, 16].

### Multidisciplinary-evaluation protocol

For patients with colorectal malignancies, contrast-enhanced computed tomography (enhanced CT) and abdominal ultrasound are deemed as standard surgical-evaluation tools. The institution’s protocol states that, if liver metastases are highly suspected, hepatic enhanced magnetic resonance imaging (enhanced MR) is routinely arranged for further investigation. Strategies for liver metastases (hepatic excision or RFA) were plotted through the discussion in the multiple-disciplinary team (MDT).

### Data-extraction protocol

The clinical, radiological, pathological data, and laboratory results were retrospectively extracted from the Electrical Medical Record System of the institution. For each patient, abdominal enhanced CT and hepatic enhanced MR were reexamined separately by two radiologists with >5 years of experience to reduce evaluation bias. Clinical data consisted of baseline information (sex, age, body mass index, diabetes, and synchronous/metachronous type). Radiological characteristics include primary-tumor location, lymph-node (LN) metastasis suspicion (regional LN >1 cm or cluster of LNs >3) from enhanced CT at diagnosis, clinical T category, existence of extra-liver metastasis, liver-metastases number, maximum lesion diameter, and lobular distribution. Pathological features include differentiation, pathological T and N category, number of lymph nodes invaded, and neurovascular invasion (NVI). Carcinoembryonic antigen (CEA) and carbohydrate antigen 19–9 (CA19–9) levels at diagnosis, before surgery, and after surgery were collected. The neutrophil–lymphocyte ratio, platelet-to-lymphocyte ratio, serum albumin (Alb), albumin–globulin ratio, aspartate aminotransferase (AST), alanine aminotransferase (ALT), and prothrombin time were extracted from the preoperative laboratory tests.

### Statistical analysis

Among the included patients, those who developed early recurrence based on the above-mentioned criteria were grouped as ER, whereas the others were grouped as non-early recurrence (NER). Statistical algorithms were used to verify the concordance between the two groups. Data distribution was evaluated using the Kolmogorov–Smirnov test. Continuous variables are presented as mean ± standard deviation (SD) if normally distributed, whereas the non-normally distributed variables are viewed as median (first-third quartile value). Categorical variables are listed as numbers (percentages). Statistical differences for categorical, continuous, and layered variables were examined using Pearson’s chi-square, Student’s *t*-test, and rank-sum test, respectively. Statistical significance between the prediction models was verified using the Mann–Whitney’s *z*-test. For individual characteristics including liver-metastases number and serum molecular markers at diagnosis, before surgery, and after surgery, we used the receiver-operating characteristic (ROC) curves to determine the optimal cut-off value for ER prediction. Alb levels <40 g/L suggest malnutrition, so we used 40 g/L as the cut-off value.

Factors with *P*-values <0.1 in univariate logistic regression or of clinical significance were eligible for the multivariate model. Predictive models for ER were formulated based on a multivariate-regression model and then used to compute ER risk scores for each patient. The area under the curve (AUC) was adopted to assess the model’s performance. An AUC of 0.5–0.7, 0.7–0.9, and >0.9 would be considered modest, good, and excellent, respectively. The Youden index was calculated to determine an ideal threshold for high risk of ER. Subsequently, a predictive nomogram was developed to visualize the aforementioned model, which was tested for discrimination, calibration, and performance against published models: Beppu *et al.* [28] developed a predictive nomogram in a comparable setting, in which synchronous disease, positive LN metastasis, number of tumors stratified at 1, 2–4, and >4, largest tumor size, extra-liver metastases, and preoperative CA19–9 >100 U/mL were included. Also, the clinical risk score (CRS) [29] has been widely adopted for the post-operative prognosis of CRLM patients and these models were used as comparison. Additionally, lift curves were used to investigate how nomogram-assisted post-operative surveillance will bring net benefit to patients in clinical practice. A two-tailed *P*-value of <0.05 was deemed statistically significant. All statistical analyses were performed using SPSS (version 25, Chicago, IL, USA) and R software (Version 3.6.2).

## Results

### Data characteristics

**Figure 1.**
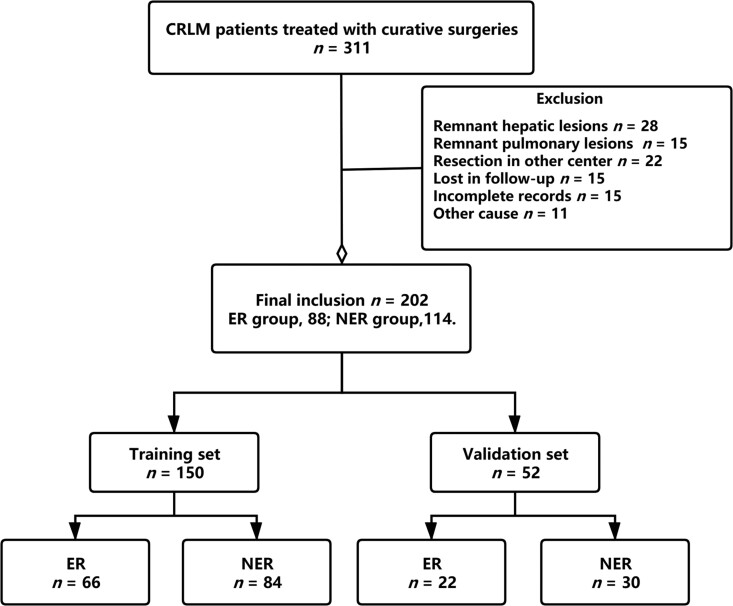
Elements of patients enrolled in this study. Three hundred and eleven CRLM patients who underwent curative surgeries between 2012 and 2019 were enrolled in this study; among them, 109 were excluded according to the exclusion criteria. The remaining 202 were randomly assigned to the training set (*n *=* *150) and validation set (*n *=* *52). There were 88 cases (43.6%) of early recurrence in the included patients. CRLM, colorectal-cancer liver metastases; ER, early recurrence; NER, non-early recurrence.

There were 311 CRLM patients who underwent surgeries for primary and metastatic tumors in the Second Affiliated Hospital of Zhejiang University School of Medicine. For criteria selection, 109 were excluded and 202 satisfied the inclusion criteria (**Figure 1**). Among them, 150 patients were randomly assigned to the training group. The rest were validated. The overall recurrence rate was 77.7% (157 out of 202 patients). Eighty-eight (43.6%) were stratified into ER, while 114 were NER. The ER rates in the training and validation groups were 44.0% and 42.3%, respectively.

**Table 1** presents the aforementioned data in the training and validation groups (full scale in **Supplementary Table 1**). Using the above-mentioned method, the cut-off values for CEA at diagnosis, before surgery, and after surgery were 100, 5, and 6 ng/mL, and the cut-off limits for CA19-9 were 320, 70, and 13 U/mL, respectively. All variables between training and validation were comparable, suggesting satisfactory concordance. Notably, post-operative CEA and CA19-9 with cut-off values of 6 ng/mL and 13 U/mL, respectively, in both wings revealed strong statistical significance. In addition, the number of liver metastases and its threshold at four were found to be statistically significant (*P *<* *0.001) in the training set.

**Table 1.** Patient characteristics in the training and validation sets

Variable	Training set				Validation set				
	Total	ER	NER	*P*	Total	ER	NER	*P*	$\bar{p}$
Age	61.4 ± 9.9	62.7 ± 10.5	60.5 ± 9.3	0.177	58.4 ± 8.6	59.1 ± 8.6	57.9 ± 8.6	0.639	0.055
Sex									
Male	108 (72.0)	44 (66.7)	64 (76.2)	0.206	32 (61.5)	14 (63.6)	18 (60)	>0.999	0.167
Female	42 (28.0)	22 (33.3)	20 (23.8)		20 (38.5)	8 (36.4)	12 (40)		
BMI	22.7 ± 3.2	23.1 ± 3.5	22.5 ± 3.0	0.289	22.2 ± 2.9	21.0 ± 2.8	23.1 ± 2.6	>0.999	0.272
Disease									
Synchronous	107 (71)	52 (78.8)	55 (65.5)	0.101	36 (69.2)	12 (54.5)	24 (80)	0.07*	0.860
Metachronous	43 (29)	14 (21.2)	29 (34.5)		16 (30.8)	10 (45.5)	6 (20)		
Colon/rectum									
Colon	94 (62.7)	48 (72.7)	46 (54.8)	0.038	39 (75.0)	16 (72.7)	23 (76.7)	0.757	0.172
Rectum	53 (35.3)	17 (25.8)	36 (42.9)		13 (25.0)	6 (27.3)	7 (23.3)		
Right/left colon									
Right	45 (30.0)	24 (36.4)	21 (25.0)	0.153	23 (44.2)	10 (45.5)	13 (43.3)	>0.999	0.088
Left	105 (70.0)	42 (63.6)	63 (75.0)		29 (55.8)	12 (54.5)	17 (56.7)		
LN suspicion									
No	27 (18)	9 (6)	18 (12)	0.279	13 (25)	2 (9.1)	11 (36.7)	0.157	0.131
Yes	109 (72.7)	51 (34)	58 (38.7)		27 (51.9)	11 (50.0)	16 (53.3)		
Unknown	14 (9.3)	6 (4)	8 (5.3)		12 (23.1)	9 (40.9)	3 (10)		
Number of metastases									
≤4	110 (73.3)	38 (57.6)	72 (85.7)	<0.001	39 (75)	13 (59.1)	26 (86.7)	0.090	0.578
>4	40 (26.7)	28 (42.4)	12 (14.3)		11 (21.2)	7 (31.8)	4 (13.3)		
Unknown					2 (3.8)	2 (9.1)			
Maximum diameter (cm)	2.5 (1.8–4.5)	2.7 (1.9–5.4)	2.2 (1.7–3.8)	0.034	2.9 (2.3–4.0)	1.7 (1.3–2.0)	1.8 (1.4–2.4)	0.283	0.718
Lobular distribution									
Monolobular	107 (71.3)	39 (59.1)	68 (81.0)	0.004	31 (59.6)	12 (54.5)	19 (63.3)	0.564	0.167
Bilobular	43 (28.7)	27 (40.9)	16 (19.0)		20 (38.5)	10 (45.5)	10 (33.3)		
Unknown					1 (1.9)		1 (3.3)		
NVI									
No	59 (39.3)	18 (27.3)	41 (48.8)	0.010	24 (46.2)	8 (36.4)	16 (53.3)	0.766	0.402
Yes	80 (53.3)	42 (63.6)	38 (45.2)		24 (46.2)	10 (45.5)	14 (46.7)		
Unknown	11 (7.3)	6 (9.1)	5 (6)		4 (7.7)	4 (18.2)			
CEA level at diagnose (ng/mL)									
≤100	130 (86.7)	50 (75.8)	80 (95.2)	0.002	37 (71.2)	15 (68.2)	22 (73.3)	0.018*	0.762
>100	20 (13.3)	16 (24.2)	4 (4.8)		15 (28.8)	7 (31.8)	8 (26.7)		
CEA level after surgery (ng/mL)									
≤6	94 (62.7)	31 (47)	63 (75)	0.001	34 (65.4)	9 (40.9)	25 (83.3)	0.003*	0.867
>6	56 (37.3)	35 (53)	21 (25)		18 (34.6)	13 (59.1)	5 (16.7)		
CA19-9 level at diagnose (U/mL)									
≤320	121 (80.7)	46 (69.7)	75 (89.3)	0.004	42 (80.8)	15 (68.2)	27 (90)	0.075	>0.999
>320	29 (19.3)	20 (30.3)	9 (10.7)		10 (19.2)	7 (31.8)	3 (10)		
CA19-9 level after surgery (U/mL)									
≤13	72 (48)	19 (28.8)	53 (63.1)	0.001	28 (53.8)	7 (31.8)	21 (70)	0.011*	0.521
>13	78 (52)	47 (71.2)	31 (36.9)		24 (46.2)	15 (68.2)	9 (30)		
Albumin (g/L)									
≤40	66 (44)	36 (54.5)	30 (35.7)	0.022	19 (36.5)	8 (36.4)	11 (36.7)	>0.999	0.416
>40	84 (56)	30 (45.5)	54 (64.3)		33 (63.5)	14 (63.6)	19 (63.3)		

Baseline information, clinical, radiological, pathological*,* and serum tests were statistically analysed*. P* represents the statistical significance between the early-recurrence and non-early-recurrence groups within each set.$\bar{p}$ represents the statistical significance between the training set and validation set. Continuous variables are presented as mean ± standard deviation (SD) if normally distributed, whereas non-normally distributed variables are shown as median (first-third quartile value). Categorical variables are listed as numbers (percentages).

ER, early recurrence; NER, non-early recurrence; BMI, body mass index; LN, lymph node; NVI, neurovascular invasion; CEA, carcinoembryonic antigen; CA19-9, carbohydrate antigen 19-9.

### Development and validation of a predictive nomogram

In the training set, univariable logistic regression was performed (full scale in **Supplementary Table 2**), in which synchronous/metachronous metastasis, colon/rectum location, lesion number, maximum diameter, lobular distribution, NVI, CEA, and CA19-9 (stratified) at diagnosis, before surgery, and post operation, respectively, and Alb level (stratified) was statistically significant. LN suspicion, for its critical role in the preoperative radiological evaluation, was included in the following multivariate test. Based on the multivariate model, lesion number, LN suspicion, NVI, colon/rectum location, Alb level, and post-operative CEA and CA19-9 were prognostic predictors (**Table 2**). The weight of each parameter was determined by regression coefficients in the multivariable model. The risk-score formula was as follows:

p=(–2.879)+(Lesions>4)×1.424+LN suspicion×1.349+NVI×1.014+rectum site×(–1.196)+(CEA level after surgery>6)×1.287+(CA19−9 level after surgery>13)×1.656+(Alb>40)×(–1.074)

**Table 2.** Univariate and multivariate logistic-regression test for included variables

Characteristic	Univariate analysis		Multivariate analysis	
	Hazard ratio (95% CI)	*P*	Hazard ratio (95% CI)	*P*
Colon/rectum		0.027		0.021
Colon	Reference		Reference	
Rectum	0.453 (0.224–0.915)		0.302 (0.104–0.809)	
LN suspicion		0.211		0.035
No	Reference		Reference	
Yes	1.759 (0.726–4.258)		3.852 (1.151–14.429)	
Number of metastases		<0.001		0.006
≤4	Reference		Reference	
>4	4.421 (2.022–9.665)		4.154 (1.547–12.042)	
Maximum diameter (cm)	1.168 (1.014–1.346)	0.032		
NVI		0.010		0.033
No	Reference		Reference	
Yes	2.518 (1.242–5.105)		2.755 (1.106–7.232)	
CEA level after surgery (ng/mL)		<0.001		0.015
≤6	Reference		Reference	
>6	3.387 (1.697–6.760)		3.623 (1.314–10.685)	
CA19-9 level after surgery (U/mL)		<0.001		<0.001
≤13	Reference		Reference	
>13	4.229 (2.115–8.458)		5.238 (1.960–15.158)	
Albumin (g/L)		0.022		0.023
≤40	Reference		Reference	
>40	0.463 (0.240–0.895)		0.341 (0.129–0.845)	

CI, confidence interval; LN, lymph node; NVI, neurovascular invasion; CEA, carcinoembryonic antigen; CA19-9, carbohydrate antigen 19-9.

A univariable logistic-regression test was performed. Variables showing significance <0.1 or were critical in evaluation will be eligible for the multivariate logistic-regression test. Independent prognostic factors via multivariate tests would be further used to develop the predictive model.

**Figure 2.**
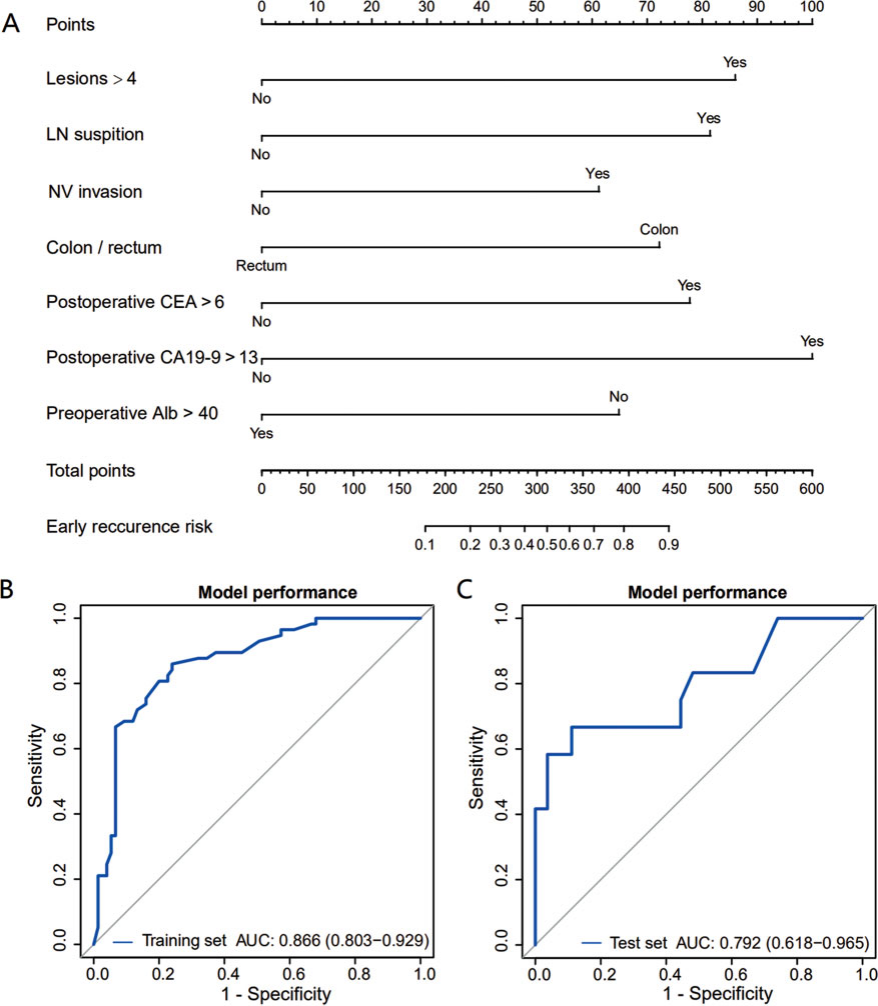
Predictive nomogram with internal and external validation. (A) The predictive model was visualized into a seven-factor nomogram to predict ER in CRLM patients after curative surgery. (B) and (C) Internal and external AUC were calculated to evaluate the performance of this model. The AUC was comparable in both sets and showed outstanding precision. CRLM, colorectal-cancer liver metastases; AUC, area under the curve; LN, lymph node; NV, neurovascular; CEA, carcinoembryonic antigen; CA19-9, carbohydrate antigen 19-9; Alb, albumin.

For each patient, the ER risk scores were calculated. After that, ROC curves were plotted for internal (training wing) and external validation (test wing) to verify the model’s performance (**Figure 2**). The AUC was 0.866 (95% confidence interval [CI], 0.803–0.929) and 0.792 (95% CI, 0.618–0.965) in the training and validation sets, respectively, indicating satisfactory performance. In the training set, the Youden value peaks with the threshold of ER risk at 0.399. Considering that timely detection of relapse during post-operative surveillance aids early intervention [30], we suggest that patients with ER probability >0.4 should be tagged as being at high risk and warrant further attention.

**Figure 3.**
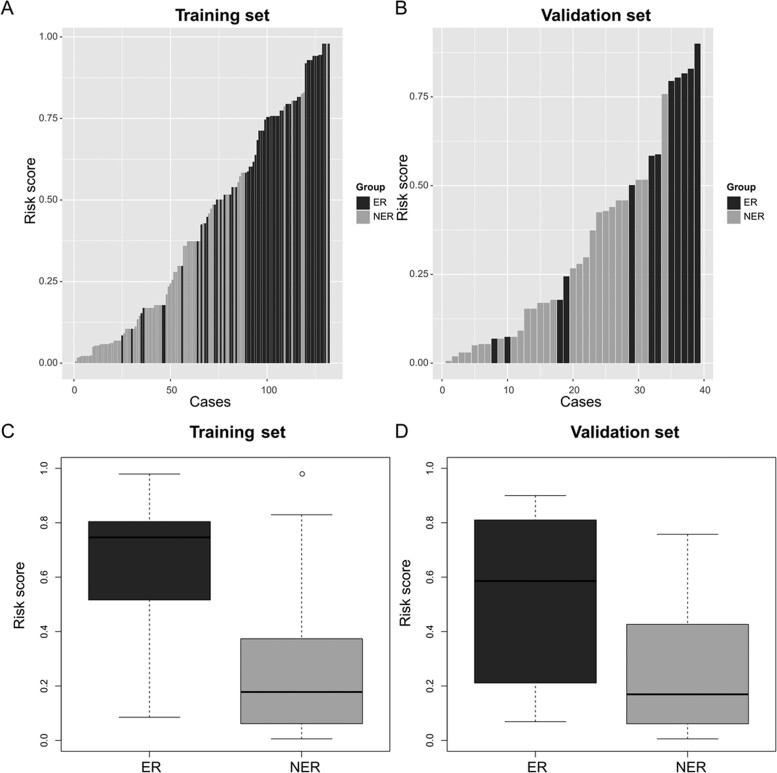
Distribution of early-recurrence-risk scores in training and validation sets. (A) and (B) Risk scores of each patient in the training and validation groups were calculated via nomogram and sorted by risk-score value. (C) and (D) In both the training and validation sets, the early-recurrence group had significantly higher risk scores (**P <* *0.01; ***P <* *0.001). ER, early recurrence; NER, non-early recurrence.

To aid its bedside utility, we used a nomogram to visualize this model. The nomogram and prediction performance via ROC curves in the internal and external validation are shown in **Figure 2**. In the descriptive analysis of the distribution of ER risk scores among the training and validation sets, the ER group had significantly higher risk scores than the NER group (*P *<* *0.001 and 0.01, respectively) (**Figure 3**).

### Calibration and assessments of the nomogram

**Figure 4.**
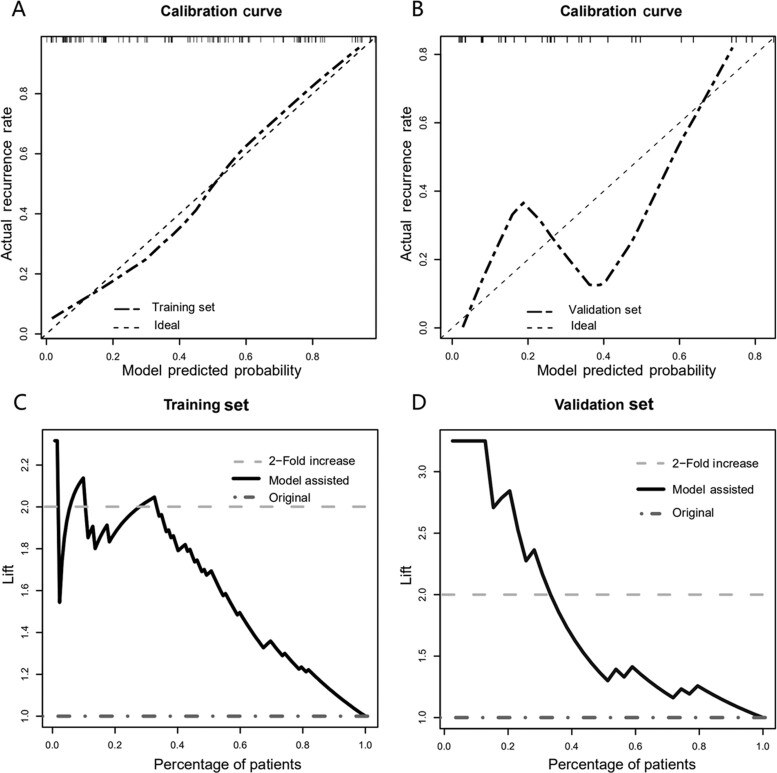
Calibration for predictive nomogram and its clinical utility. Calibration curves were used to reflect the concordance between nomogram prediction and actual distribution. The 45-degree dashed line represents an ideal prediction. (A) In the training set, it showed excellent calibration. (B) In the validation set, owing to the limited sample pool, there were some wobbles in the calibration curve but it still drifted to the dashed line. Lift curves in training (C) and validation (D) sets. In both sets, a lift value of 2 was achieved in roughly the top 30% of cases. In clinical interpretation, this meant a 2-fold detection rate in the post-operative surveillance in the top 30 high-risk patients with model-aid strategies.

Calibration curves using the bootstrap method (1,000 times) were plotted (**Figure 4A** and **B**). In the training set, the prediction curve showed perfect alignment with the dashed line, suggesting agreement between the prediction and actual outcome. In the validation set, the prediction curve showed a wobbly feature likely resulting from a limited sample, but again did not drift much from the dashed line.

Using our data, the AUC value with Beppu’s and CRS model in the whole data set was 0.686 and 0.654, respectively, while the AUC with our model was 0.857 (**Supplementary Figure 1**). Using the *z*-test, our nomogram significantly surpassed these prediction models in the training set (*P *<* *0.001).

We further investigated how this nomogram would benefit the detection rate during post-operative surveillance. Lift curves in both groups were drawn to represent the increase in the recall rate when the model was used (**Figure 4C** and **D**). The dark-grey horizontal line (*y* = 1) represents the original condition, whereas the lift curve (black) represents the increased recall rate. Definitively, in both groups, the dark-grey line fell below the lift curve for the entire duration, suggesting that, in all cases, using the nomogram will provide benefit in comparison to not using it. In addition, when roughly the top 30% of high-risk patients were selected, a lift value of 2 was achieved (**Figure 4**). In clinical interpretation, this meant a 2-fold increase in the detection for the top 30% high-risk patients using the model-aid post-operative surveillance.

## Discussion

A bedside clinical predictive system is urgently needed to predict ER in CRLM patients after curative surgeries to optimize individually tailored post-operative surveillance strategies. In the present study, we developed and validated a seven-factor nomogram that discriminates ER and NER patients with remarkable accuracy in both the training and validation groups. All variables were acquired in the routine CRLM treatment, which added to the cost-effectiveness of our nomogram. Using this nomogram, each patient will have an individualized risk score indicating the probability of developing ER.

The time definition of ‘early recurrence’ in CRC has not been universally established for all stages of CRC, as prognosis differs greatly among different clinical stages [17, 18, 31, 32]. Clearly, due to the nature of terminal stage colorectal malignancy, a shorter interval should be considered. In the present study, ER was defined as CRC recurring within 6 months after curative surgery based on previous literature [13, 14]. Indeed, in our study, the ER rate was 43.6% and the overall recurrence rate was 77.7%. Through literature research, our data were comparable to those of other studies [17, 18, 28].

Our presentation highlighted the role of liver-metastases number, radiological LN involvement, NVI, colon/rectum location, post-operative serum molecular markers, and preoperative nutrition levels. In logistic regression, more than four liver lesions showed the strongest statistical correlation with ER. Indeed, multiple intra-liver metastases not only represent worse tumor behavior, but also are surgically challenging. When multiple metastases are present, surgeons tend to resect ‘the first’ instead of the metastasis in the target area, causing remnant lesions. Besides, to remove all lesions, lobular or even extended lobe excision was used, which removes more liver volume [33] and provides little room for salvageability [34].

The utility of preoperative serum molecular markers in CRC prognostic prediction has drawn great attention, but prediction using post-operative markers has been less frequently discussed. In our study, post-operative CEA >6 ng/mL revealed very strong predictive potential (OR = 3.62; 95% CI, 1.31–10.68) over preoperative stratified CEA. Interestingly, post-operative CEA levels >6 ng/mL within 1 month showed incredible predictability. Lin *et al.* [35] also highlighted the significance of post-operative CEA in relapse prediction. Araujo *et al.* [36] reported a post-operative CEA level of 15 ng/mL to be effective in predicting recurrence.

On the other hand, post-operative CA19-9 has rarely been discussed as a prognostic factor in CRLM, but still showed great potential for predicting CRC recurrence [37, 38]. In this study, a post-operative CA19-9 level of 13 U/mL, which fell within the normal range, showed a strong correlation with ER (OR = 5.23; 95% CI, 1.96–15.16). Again, the power of post-operative CA19-9 was greater than the preoperative levels. These findings suggest that, even if post-operative CA19-9 is within the normal limits, it still warrants further stratification. Thus, in the present study, we used post-operative CA19-9 and CEA levels to predict ER in CRLM patients.

Serum Alb is a reliable factor that is reflective of a patient’s nutritional status [39]. The Alb level was originally adopted in intensive-care medicine to identify critical illness [40] and is now showing utility in prognosis prediction [41–43]. A serum Alb level <40 g/L suggests malnutrition [39] and was used for clinical stratification. In our study, a higher Alb level was a strong protective factor (OR = 0.34; 95% CI, 0.13–0.85). A descending Alb level not only mirrors the patient’s deteriorating condition [40], but also suggests a compromise in his immunity [44]. Our study integrated nutrition factors into the nomogram for recurrence prediction. Based on the results, our work stressed the critical role of balancing patients’ nutrition in lowering ER risk.

Moreover, colon/rectum distribution revealed statistical significance in the multivariable tests, where rectal location is a protective factor. The impact of colon/rectum location on prognosis has been widely discussed with differing opinions: Kuhry *et al.* [45] reported neutral recurrence in the colon and rectum, whereas Kishiki *et al.* [46] reported that the rectal site was a risk factor, while, in the work by Fields *et al.* [47], rectal location had a protective influence and was associated with better survival. Supporting his study, in our final model, rectal site has an OR of 0.30 (95% CI 0.10–0.81), suggesting a strong protective effect.

In contrast to those aiming at early stages that have shown higher accuracy, nomograms for recurrence after curative surgery in CRLM patients with usability and accuracy have not been satisfactory due to the complexity of CRLMs [7, 17, 28, 48, 49]. Our nomogram yielded an AUC of 0.866 and 0.792 for internal and external validation, respectively. Beppu’s model [28] was one of the very few that was externally validated, and the AUC was 0.59 [49]. CRS is another model that cannot be neglected, as it has been most widely used for post-operative recurrence. When both models were put to the test in our data set, the AUC values were significantly outperformed (both *P *<* *0.001) by our prediction model (**Supplementary Figure 1**). To the best of our knowledge, our nomogram exhibited by far the highest precision.

The gravity of any clinical-oriented nomogram determines whether this nomogram-assisted decision-making will bring net benefit to patients in clinical practice. In our study, the optimal threshold for ER probability was 0.4. It is fair to argue that patients with an ER risk >0.4 should be viewed as being at high risk and warrant further attention.

Since the prognosis of CRLM patients after curative surgeries varies dramatically, routine post-operative surveillance is suboptimal for being either coarse for ER patients or financially draining for NER patients. Instead, post-operative surveillance should be more individualized [50] not only in terms of the check-up interval, but also in surveillance means. Thus, we discussed how utilizing this nomogram will benefit timely relapse detection during post-operative surveillance. The lift curve is most frequently used in marketing to evaluate whether a selective model aids in the management strategies. For instance, a blind pick-up in a pool of customers containing 20% of active responders yields an ∼20% response rate (RR). When active responders are selectively picked via a classifier, a higher RR will be achieved. Using our nomogram as a classifier, a >2-fold increase in the detection (response) rate was seen in the top 30% of patients who were likely to develop ER (responders). In clinical interpretation, we expect a great increase in the detection rate when highly selective follow-up strategies are provided for high-risk patients using our model. Bhattacharyya *et al.* [51] reported a 3-month lag in detecting relapse using the sole method, while a combination of serum tumor markers and radiology boosts surveillance efficiency dramatically. Based on our findings, we recommend a more intensive surveillance protocol (e.g. hepatic MR and abdominal enhanced CT every other month, serum-tumor-marker tests every month, and introduction of PET-CT when post-operative recurrence is suspected) in high-risk populations.

Our study has the following strengths: relatively large sample size, comprehensive inclusion of multidisciplinary factors, novel integration of post-operative serum markers and nutrition factors to the nomogram, cost-friendliness, and, above all, superior performance in ER forecasting. When the nomogram is used to aid individualized post-operative surveillance, an increased detection rate is expected. Our present study has one limitation: this is a single-centered retrospective study in which patients were of the same ethnicity and Ras/Braf information was not enrolled due to missing data. Therefore, it is ideal that our research be further validated in a multi-centered prospective research ideally documenting Ras/Braf data.

## Conclusion

We developed and validated a powerful bedside nomogram to predict ER in CRLM patients after curative surgeries. The nomogram yielded superior accuracy compared to other models and could aid in decision-making in post-operative surveillance.

## Supplementary Data

Supplementary data is available at *Gastroenterology Report* online.

## Authors’ contributions

S.D. and Y.Y. designed the study. S.D. and X.K. collected the data. Y.Y. and X.K. performed statistical analysis. J.L. provided quality control. K.D. supervised the conduction of the research.

## Funding

This work was supported by the Key Technology Research and Development Program of Zhejiang Province [No.2017C03017], the National Natural Science Foundation of China [81672916, 11932017, 81802750], the Natural Science Foundation of Zhejiang Province [LQ20H180014 to Y.Y.], the China Postdoctoral Science Foundation [2019M652117 to Y.Y.], and the Natural Science Foundation of Zhejiang Province [LBY20H160002].

## Supplementary Material

goaa092_Supplementary_Data
