# Supplementary material for: A predictive model for early recurrence of colorectal-cancer liver metastases based on clinical parameters
Source: Gastroenterol Rep (Oxf). 2021 Jan 26;9(3):241–51. doi: 10.1093/gastro/goaa092 (PMC8309687; doi:10.1093/gastro/goaa092)
Supplement: goaa092_Supplementary_Data [file goaa092_supplementary_data.zip › 2020-350 Supplementary Tables and Figure.docx]

| Supplementary Table 1. Patient characteristics in training and validation sets (whole scale) | | | | | | | | | | | | | |
| --- | --- | --- | --- | --- | --- | --- | --- | --- | --- | --- | --- | --- | --- |
| Variable | Training set | | | | |  | Validation set | | | | |  |  |
|  | Total | ER | NER |  | *P* |  | Total | ER | NER |  | *P* |  | *‾P* |
| Age | 61.4±9.9 | 62.7±10.5 | 60.5±9.3 |  | 0.177 |  | 58.4±8.6 | 59.1±8.6 | 57.9±8.6 |  | 0.639 |  | 0.055 |
| Sex |  |  |  |  |  |  |  |  |  |  |  |  |  |
| Male | 108 (72.0) | 44 (66.7) | 64 (76.2) |  | 0.206 |  | 32 (61.5) | 14 (63.6) | 18 (60) |  | >0.999 |  | 0.167 |
| Female | 42 (28.0) | 22 (33.3) | 20 (23.8) |  |  |  | 20 (38.5) | 8 (36.4) | 12 (40) |  |  |  |  |
| BMI | 22.7±3.2 | 23.1±3.5 | 22.5±3.0 |  | 0.289 |  | 22.2±2.9 | 21.0±2.8 | 23.1±2.6 |  | >0.999 |  | 0.272 |
| Disease |  |  |  |  |  |  |  |  |  |  |  |  |  |
| Synchronous | 107 (71) | 52 (78.8) | 55 (65.5) |  | 0.101 |  | 36 (69.2) | 12 (54.5) | 24 (80) |  | 0.070 |  | 0.860 |
| Metachronous | 43 (29) | 14 (21.2) | 29 (34.5) |  |  |  | 16 (30.8) | 10 (45.5) | 6 (20) |  |  |  |  |
| Primary site |  |  |  |  | 0.049 |  |  |  |  |  | 0.500 |  | 0.155 |
| Ileocecal | 8 (5.3) | 4 (6.1) | 4 (4.8) |  |  |  | 1 (1.9) | 1 (4.5) | 0 (0) |  |  |  |  |
| Ascending | 22 (14.7) | 11 (16.7) | 11 (13.1) |  |  |  | 9 (17.3) | 7 (31.8) | 2 (6.7) |  |  |  |  |
| Hepatic | 6 (4.0) | 3 (4.5) | 3 (3.6) |  |  |  | 8 (15.4) | 1 (4.5) | 7 (23.3) |  |  |  |  |
| Transverse | 9 (6.0) | 6 (9.1) | 3 (3.6) |  |  |  | 4 (7.7) | 0 (0) | 4 (13.3) |  |  |  |  |
| Splenic | 1 (0.7) | 1 (1.5) | 0 (0) |  |  |  | 0 (0) | 0 (0) | 0 (0) |  |  |  |  |
| Descending | 6 (4.0) | 2 (3) | 4 (4.8) |  |  |  | 3 (5.8) | 1 (4.5) | 2 (6.7) |  |  |  |  |
| Sigmoid | 42 (28.0) | 21 (31.8) | 21 (25) |  |  |  | 13 (25.0) | 5 (22.7) | 8 (26.7) |  |  |  |  |
| Rectum | 53 (35.3) | 17 (25.8) | 36 (42.9) |  |  |  | 13 (25.0) | 6 (27.3) | 7 (23.3) |  |  |  |  |
| Unknown | 3 (2.0) | 1 (1.5) | 2 (2.4) |  |  |  |  |  |  |  |  |  |  |
| Colon/Rectum |  |  |  |  |  |  |  |  |  |  |  |  |  |
| Colon | 94 (62.7) | 48 (72.7) | 46 (54.8) |  | 0.038 |  | 39 (75.0) | 16 (72.7) | 23 (76.7) |  | 0.757 |  | 0.172 |
| Rectum | 53 (35.3) | 17 (25.8) | 36 (42.9) |  |  |  | 13 (25.0) | 6 (27.3) | 7 (23.3) |  |  |  |  |
| Right/ Left Colon |  |  |  |  |  |  |  |  |  |  |  |  |  |
| Right | 45 (30.0) | 24 (36.4) | 21 (25.0) |  | 0.153 |  | 23 (44.2) | 10 (45.5) | 13 (43.3) |  | >0.999 |  | 0.088 |
| Left | 105 (70.0) | 42 (63.6) | 63 (75.0) |  |  |  | 29 (55.8) | 12 (54.5) | 17 (56.7) |  |  |  |  |
| Clinical T4 category |  |  |  |  |  |  |  |  |  |  |  |  |  |
| No | 110 (73.3) | 27 (40.9) | 38 (45.2) |  | 0.622 |  | 16 (30.8) | 5 (22.7) | 11 (36.7) |  | 0.535 |  | 0.242 |
| Yes | 40 (26.7) | 39 (59.1) | 46 (54.8) |  |  |  | 32 (61.5) | 14 (63.6) | 18 (60) |  |  |  |  |
| Unknown |  |  |  |  |  |  | 4 (7.7) | 3 (13.6) | 1 (3.3) |  |  |  |  |
| LN suspicion |  |  |  |  |  |  |  |  |  |  |  |  |  |
| No | 27 (18) | 9 (6) | 18 (12) |  | 0.279 |  | 13 (25) | 2 (9.1) | 11 (36.7) |  | 0.157 |  | 0.131 |
| Yes | 109 (72.7) | 51 (34) | 58 (38.7) |  |  |  | 27 (51.9) | 11 (50.0) | 16 (53.3) |  |  |  |  |
| Unknown | 14 (9.3) | 6 (4) | 8 (5.3) |  |  |  | 12 (23.1) | 9 (40.9) | 3 (10) |  |  |  |  |
| Extra-hepatic metastasis | | | |  |  |  |  |  |  |  |  |  |  |
| Negative | 133 (88.7) | 55 (83.3) | 78 (92.9) |  | 0.118 |  | 46 (88.5) | 17 (77.3) | 29 (96.7) |  | 0.072 |  | >0.999 |
| Positive | 16 (10.7) | 10 (15.2) | 6 (7.1) |  |  |  | 6 (11.5) | 5 (22.7) | 1 (3.3) |  |  |  |  |
| Unknown |  | 1(1.5) |  |  |  |  |  |  |  |  |  |  |  |
| Number of metastases | 2 (1-4) | 2 (1-5) | 1 (1-2) |  | <0.001 |  | 2 (1-3) | 2 (1-5) | 1 (1-2) |  | 0.076 |  | 0.679 |
| ≤4 | 110 (73.3) | 38 (57.6) | 72 (85.7) |  | <0.001 |  | 39 (75) | 13 (59.1) | 26 (86.7) |  | 0.090 |  | 0.578 |
| >4 | 40 (26.7) | 28 (42.4) | 12 (14.3) |  |  |  | 11 (21.2) | 7 (31.8) | 4 (13.3) |  |  |  |  |
| Unknown |  |  |  |  |  |  | 2 (3.8) | 2 (9.1) |  |  |  |  |  |
| Maximum diameter (cm) | 2.5 (1.8-4.5) | 2.7 (1.9-5.4) | 2.2 (1.7-3.8) |  | 0.034 |  | 2.9 (2.3-4.0) | 1.7 (1.3-2.0) | 1.8 (1.4-2.4) |  | 0.283 |  | 0.718 |
| Lobular distribution |  |  |  |  |  |  |  |  |  |  |  |  |  |
| Monolobular | 107 (71.3) | 39 (59.1) | 68 (81.0) |  | 0.004 |  | 31 (59.6) | 12 (54.5) | 19 (63.3) |  | 0.564 |  | 0.167 |
| Bilobular | 43 (28.7) | 27 (40.9) | 16 (19.0) |  |  |  | 20 (38.5) | 10 (45.5) | 10 (33.3) |  |  |  |  |
| Unknown |  |  |  |  |  |  | 1 (1.9) |  | 1 (3.3) |  |  |  |  |
| Pathological T category |  |  |  |  |  |  |  |  |  |  |  |  |  |
| 2 | 7 (4.7) | 2 (3.0) | 5 (6.0) |  | 0.333 |  | 4 (7.7) | 1 (4.5) | 3 (10) |  | 0.038 |  | 0.743 |
| 3 | 82 (54.7) | 35 (53.0) | 47 (56.0) |  |  |  | 26 (50.0) | 7 (31.8) | 19 (63.3) |  |  |  |  |
| 4 | 50 (33.3) | 24 (36.4) | 26 (31.0) |  |  |  | 19 (36.5) | 11 (50.0) | 8 (26.7) |  |  |  |  |
| Unknown | 11 (8.3) | 5 (7.6) | 6 (7.2) |  |  |  | 3 (5.8) | 3 (13.6) | 0 (0) |  |  |  |  |
| LN Invaded | 1 (0-3.8) | 1 (0-4) | 1 (0-3) |  | 0.897 |  | 1 (1-4) | 1 (0-2) | 1 (0-2) |  | 0.025 |  | 0.520 |
| LN found | 14 (11.3-18) | 14 (12.0-17.0) | 14 (11-18) |  | 0.856 |  | 13 (11-18) | 14 (12-19) | 13 (12-16) |  | 0.602 |  | 0.958 |
| NVI |  |  |  |  |  |  |  |  |  |  |  |  |  |
| No | 59 (39.3) | 18 (27.3) | 41 (48.8) |  | 0.010 |  | 24 (46.2) | 8 (36.4) | 16 (53.3) |  | 0.766 |  | 0.402 |
| Yes | 80 (53.3) | 42 (63.6) | 38 (45.2) |  |  |  | 24 (46.2) | 10 (45.5) | 14 (46.7) |  |  |  |  |
| Unknown | 11 (7.3) | 6 (9.1) | 5 (6) |  |  |  | 4 (7.7) | 4 (18.2) |  |  |  |  |  |
| CEA level at diagnose (ng/mL) | 28.4 (9.0-95.9) | 35.5 (10.1-95.9) | 25.4 (7.7-54.9) |  | 0.331 |  | 29.9 (7.7-129.0) | 11.3 (3.8-39.1) | 16.6 (6.5-38.1) |  | 0.130 |  | 0.185 |
| ≤100 | 130 (86.7) | 50 (75.8) | 80 (95.2) |  | 0.002 |  | 37 (71.2) | 15 (68.2) | 22 (73.3) |  | 0.018 |  | 0.762 |
| >100 | 20 (13.3) | 16 (24.2) | 4 (4.8) |  |  |  | 15 (28.8) | 7 (31.8) | 8 (26.7) |  |  |  |  |
| CEA level before surgery (ng/mL) | 10.1 (4.4-33.8) | 11.4 (5.6-36.5) | 7.3 (3.5-32.5) |  | 0.095 |  | 12.1 (4.7-83.4) | 5.7 (3.5-11.7) | 6.0 (3.7-16.8) |  | 0.162 |  | 0.226 |
| ≤5 | 46 (30.7) | 12 (18.2) | 34 (40.5) |  | 0.005 |  | 16 (30.8) | 6 (27.3) | 10 (33.3) |  | 0.763 |  | 0.859 |
| >5 | 101 (67.3) | 52 (78.8) | 49 (58.3) |  |  |  | 32 (61.5) | 14 (63.6) | 18 (60) |  |  |  |  |
| Unknown | 3 (2) | 2 (3) | 1 (1.2) |  |  |  | 4 (7.7) | 2 (9.1) | 2 (6.7) |  |  |  |  |
| CEA level after surgery (ng/mL) | 4.3 (2.1-55.9) | 6.6 (2.9-33.9) | 3.3 (1.7-6.1) |  | 0.055 |  | 4.0 (2.2-8.9) | 6.1 (2.9-36.6) | 5.0 (2.5-15.3) |  | 0.311 |  | 0.612 |
| ≤6 | 94 (62.7) | 31 (47) | 63 (75) |  | 0.001 |  | 34 (65.4) | 9 (40.9) | 25 (83.3) |  | 0.003 |  | 0.867 |
| >6 | 56 (37.3) | 35 (53) | 21 (25) |  |  |  | 18 (34.6) | 13 (59.1) | 5 (16.7) |  |  |  |  |
| CA19-9 level at diagnose (U/mL) | 38.1 (10.2-238.9) | 49.7 (7.4-464.3) | 29.9 (11.3-141.5) |  | 0.039 |  | 44.3 (13.0-258.5) | 43.1 (15.5-114.3) | 21.1 (13.1-140.9) |  | 0.140 |  | 0.366 |
| ≤320 | 121 (80.7) | 46 (69.7) | 75 (89.3) |  | 0.004 |  | 42 (80.8) | 15 (68.2) | 27 (90) |  | 0.075 |  | >0.999 |
| >320 | 29 (19.3) | 20 (30.3) | 9 (10.7) |  |  |  | 10 (19.2) | 7 (31.8) | 3 (10) |  |  |  |  |
| CA19-9 level before surgery (U/mL) | 14.6 (6.0-68.5) | 18.3 (4.7-128.6) | 13.2 (6.5-40.9) |  | 0.104 |  | 22.9 (6.4-159.2) | 18.3 (11.0-40.1) | 13.1 (9.0-45.3) |  | 0.048 |  | 0.089 |
| ≤70 | 111 (74) | 42 (64.6) | 69 (82.1) |  | 0.023 |  | 32 (61.5) | 12 (54.5) | 20 (66.7) |  | 0.537 |  | 0.267 |
| >70 | 37 (24.7) | 22 (33.3) | 15 (17.9) |  |  |  | 16 (30.8) | 8 (36.4) | 8 (26.7) |  |  |  |  |
| Unknown | 2 (1.3) | 2 (3) | 0 (0) |  |  |  | 4 (7.7) | 2 (9.1) | 2 (6.7) |  |  |  |  |
| CA19-9 level after surgery (U/mL) | 14.4 (4.7-57.2) | 22.8 (10.4-57.2) | 9.3 (4.4-20.3) |  | 0.010 |  | 11.3 (3.6-41.6) | 22.8 (12.3-57.2) | 16.8 (5.9-24.5) |  | 0.278 |  | 0.612 |
| ≤13 | 72 (48) | 19 (28.8) | 53 (63.1) |  | 0.001 |  | 28 (53.8) | 7 (31.8) | 21 (70) |  | 0.011 |  | 0.521 |
| >13 | 78 (52) | 47 (71.2) | 31 (36.9) |  |  |  | 24 (46.2) | 15 (68.2) | 9 (30) |  |  |  |  |
| Albumin (g/L) | 40.8±4.5 | 39.9±4.4 | 41.5±4.5 |  | 0.033 |  | 41.9±4.5 | 41.7±3.8 | 41.7±3.8 |  | 0.872 |  | 0.180 |
| ≤40 | 66 (44) | 36 (54.5) | 30 (35.7) |  | 0.022 |  | 19 (36.5) | 8 (36.4) | 11 (36.7) |  | >0.999 |  | 0.416 |
| >40 | 84 (56) | 30 (45.5) | 54 (64.3) |  |  |  | 33 (63.5) | 14 (63.6) | 19 (63.3) |  |  |  |  |
| Albumin/Globulin |  |  |  |  |  |  |  |  |  |  |  |  |  |
| ≤1.5 | 101 (67.3) | 49 (74.2) | 52 (61.9) |  | 0.112 |  | 28 (53.8) | 13 (59.1) | 15 (50.0) |  | 0.581 |  | 0.095 |
| >1.5 | 49 (32.7) | 17 (25.8) | 32 (38.1) |  |  |  | 24 (46.2) | 9 (40.9) | 15 (50.0) |  |  |  |  |
| AST (U/L) | 25.0 (21.0-31.4) | 25 (22-33.5) | 25.5 (20.0-32.3) |  | 0.419 |  | 26.5 (21.8-35.3) | 23.5 (20.0-32.8) | 25.0 (23.0-33.3) |  | 0.348 |  | 0.081 |
| ALT (U/L) | 21.0 (16.0-28.9) | 20.5 (16-35.8) | 23 (16.0-31.0) |  | 0.547 |  | 21 (15.6-30.3) | 17.5 (15.3-30.5) | 23.0 (16.0-31.8) |  | 0.528 |  | 0.511 |
| Neutrophil vs Lymphocyte | 2.1 (1.4-3.0) | 1.9 (1.3-2.7) | 2.2 (1.5-3.3) |  | 0.284 |  | 2.1 (1.6-2.8) | 2.1 (1.7-2.8) | 2.0 (1.3-2.9) |  | 0.150 |  | 0.610 |
| Platelet vs Lymphocyte | 120.3 (88.6-159.8) | 112.4 (87.1-142.5) | 123.4 (92.5-162.3) |  | 0.235 |  | 118.4 (99.7-162.0) | 112.8 (72.7-132.8) | 113.0 (73.0-146.4) |  | 0.901 |  | 0.621 |
| Abbreviation: ER, early recurrence; NER, non-early recurrence; BMI, Body mass index; LN, Lymph node; NVI, Neurovascular invasion; CEA, Carcinoembryonic antigen; CA19-9, Carbohydrate antigen 19-9; AST, Aspartate aminotransferase; ALT, Alanine aminotransferase.  *‾P*: statistical significance between the training set and validation set; Continuous variables were presented as mean±standard deviation (SD) if normally distributed, whereas non-normally distributed variables are shown as median (first-third quartile value). Categorical variables are listed as numbers (percentages). | | | | | | | | | | | | | |

Supplement Table 2: Patients’ clinical parameters in the training and validation group. *P* represents the statistical significance between the early recurrence and non-early recurrence groups within each set.‾*P* represents the statistical significance between the training set and validation set. Continuous variables were presented as mean±standard deviation (SD) if normally distributed, whereas non-normally distributed variables are shown as median (first-third quartile value). Categorical variables are listed as numbers (percentages).

Supplement Table 2: Univariate logistic regression test for included variables (whole scale)

| **Included variable** |  | **Hazard ratio (95% CI)** | |  | ***P*** |
| --- | --- | --- | --- | --- | --- |
| **Age** |  | **1.023 (0.989-1.059)** |  | | **0.121** |
| **Sex**  **Male** |  | **Reference** |  | | **0.199** |
| **Female** |  | **1.600 (0.781-3.277)** |  | |  |
| **BMI** |  | **1.057 (0.955-1.169)** |  | | **0.288** |
| **Disease**  **Synchronous** |  | **Reference** |  | | **0.076** |
| **Metachronous** |  | **0.511 (0.243-1.072)** |  | |  |
| **Colon/Rectum** |  |  |  | | **0.027** |
| **Colon** |  | **Reference** |  | |  |
| **Rectum** |  | **0.453 (0.224-0.915)** |  | |  |
| **Right/Left** |  |  |  | | **0.133** |
| **Right** |  | **Reference** |  | |  |
| **Left** |  | **0.583 (0.298-1.179)** |  | |  |
| **Clinical T4 category** |  |  |  | | **0.595** |
| **No** |  | **Reference** |  | |  |
| **Yes** |  | **1.193 (0.622-2.291)** |  | |  |
| **LN suspicion**  **No** |  | **Reference** |  | | **0.211** |
| **Yes** |  | **1.759 (0.726-4.258)** |  | |  |
| **Extra-hepatic metastasis**  **Negative** |  | **Reference** |  | | **0.115** |
| **Positive** |  | **2.364 (0.811-6.886)** |  | |  |
| **Number of metastases** |  |  |  | |  |
| **≤4** |  | **Reference** |  | |  |
| **>4** |  | **4.421 (2.022-9.665)** |  | | **<0.001** |
| **Max diameter (cm)** |  | **1.168 (1.014-1.346)** |  | | **0.032** |
| **Distribution**  **Monolobular** |  | **Reference** |  | | **0.004** |
| **Bilobular** |  | **2.942 (1.141-6.124)** |  | |  |
| **Pathological T category** |  |  |  | |  |
| **2** |  | **Reference** | |  |  |
| **3** |  | **0.800 (0.044-14.643)** | |  | **0.880** |
| **4** |  | **1.489 (0.130-17.088)** | |  | **0.749** |
| **Number of LN invaded** |  | **1.008 (0.898-1.131)** | |  | **0.897** |
| **Number of LN found** |  | **0.995 (0.939-1.053)** | |  | **0.856** |
| **NVI**  **No** |  | **Reference** | |  | **0.010** |
| **Yes** |  | **2.518 (1.242-5.105)** | |  |  |
| **CEA level at diagnose (ng/mL)** |  |  | |  | **0.002** |
| **≤100** |  | **Reference** | |  |  |
| **>100** |  | **6.400 (2.024-20.237)** | |  |  |
| **CEA level before surgery (ng/mL)** |  |  | |  | **0.005** |
| **≤5** |  | **Reference** | |  |  |
| **>5** |  | **3.007 (1.399-6.462)** | |  |  |
| **CEA level after surgery (ng/mL)** |  |  | |  | **0.001** |
| **≤6** |  | **Reference** | |  |  |
| **>6** |  | **3.387 (1.697-6.760)** | |  |  |
| **CA19-9 level at diagnose (U/mL)** |  |  | |  | **0.004** |
| **≤320** |  | **Reference** | |  |  |
| **>320** |  | **3.623 (1.521-8.632)** | |  |  |
| **CA19-9 level before surgery (U/mL)** |  |  | |  | **0.023** |
| **≤70** |  | **Reference** | |  |  |
| **>70** |  | **2.410 (1.127-5.153)** | |  |  |
| **CA19-9 level after surgery (U/mL)** |  |  | |  | **0.001** |
| **≤13** |  | **Reference** | |  |  |
| **>13** |  | **4.229 (2.115-8.458)** | |  |  |
| **Albumin (g/L)** |  |  | |  | **0.022** |
| **≤40** |  | **Reference** | |  |  |
| **>40** |  | **0.463 (0.240-0.895)** | |  |  |
| **AST (U/L)** |  | **1.006 (0.992-1.021)** | |  | **0.419** |
| **ALT (U/L)** |  | **1.004 (0.991-1.017)** | |  | **0.547** |
| **Neutrophil vs. Lymphocyte** |  | **0.912 (0.772-1.079)** | |  | **0.284** |
| Abbreviations: CI, confidence interval; BMI, body mass index; LN, lymph node; NVI, neurovascular invasion; CEA, carcinoembryonic antigen; CA19-9, Carbohydrate antigen 19-9; AST, aspartate aminotransferase; ALT, alanine aminotransferase. | | | | | |

A univariable logistic regression test was performed. Statistical significance, HRs, and their 95% confidence intervals were presented.

Supplementary Fig. 1: AUC with Beppu’s and CRS models in the whole dataset.

(Beppu’s nomogram and CRS model were used for comparison. Using our dataset as validation, the AUC value (Jasper) with Beppu’s model (A) and CRS model (B) was 0.686 and 0.654, respectively. The AUC for our model (orange line) in the whole dataset was 0.857. Using the *z*-test, our model significantly outperformed the two models in early recurrence prediction (A and B, both *P* < 0.001). AUC, area under the curve; CRS, clinical risk score.
