# Supplementary figures and images for: A predictive model for early recurrence of colorectal-cancer liver metastases based on clinical parameters
Source: Gastroenterol Rep (Oxf). 2021 Jan 26;9(3):241–51. doi: 10.1093/gastro/goaa092 (PMC8309687; doi:10.1093/gastro/goaa092)

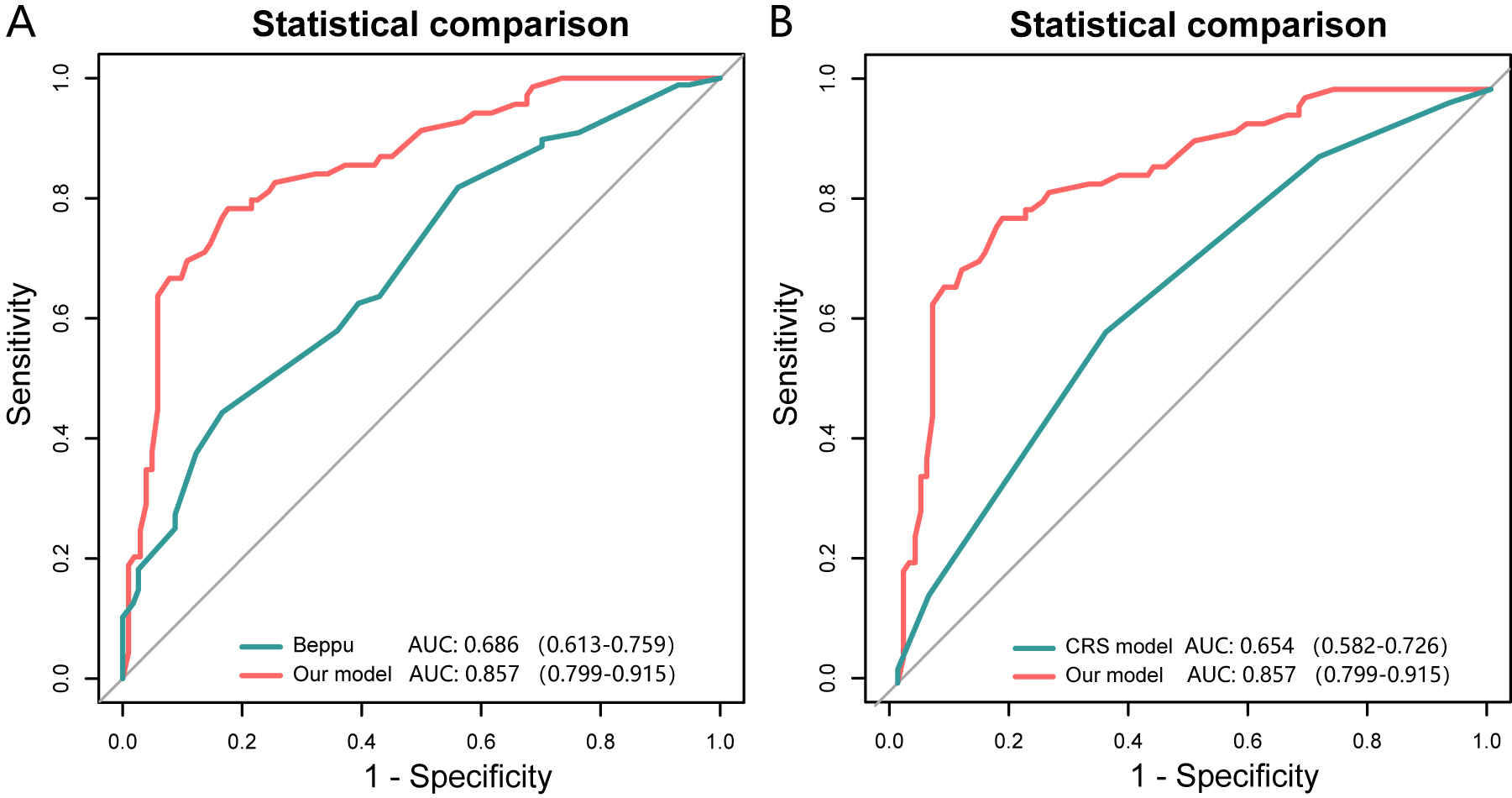

Supplement: goaa092_Supplementary_Data [file goaa092_supplementary_data.zip › 2020-350 Supplement Fig 1.jpg]
